# Integrated Fecal Microbiome and Serum Metabolomics Analysis Reveals Abnormal Changes in Rats with Immunoglobulin A Nephropathy and the Intervention Effect of Zhen Wu Tang

**DOI:** 10.3389/fphar.2020.606689

**Published:** 2021-01-27

**Authors:** Jicheng Li, Yiwen Cao, Ruirui Lu, Honglian Li, Yu Pang, Hongxin Fu, Guoxing Fang, Qiuhe Chen, Bihao Liu, Junbiao Wu, Yuan Zhou, Jiuyao Zhou

**Affiliations:** ^1^ Department of Pharmacology, School of Pharmaceutical Sciences, Guangzhou University of Chinese Medicine, Guangzhou, China; ^2^ Department of Urology, The Sixth Affiliated Hospital of Sun Yat-Sen University, Guangdong, China; ^3^ Guangdong Institute of Gastroenterology, Sun Yat-Sen University, Guangzhou, China; ^4^ The Second Affiliated Hospital, Guangzhou University of Chinese Medicine, Guangzhou, China

**Keywords:** kidney injury, immune inflammation, metabolism, gut microbiota, Zhen Wu Tang, immunoglobulin A nephropathy

## Abstract

Immunoglobulin A nephropathy (IgAN), an autoimmune renal disease with complicated pathogenesis, is one of the principal reasons for end-stage renal disease in the clinic. Evidence has linked apparent alterations in the components of the microbiome and metabolome to renal disease in rats. However, thus far, there is insufficient evidence that supports the potential relationship between gut microbiome, circulating metabolites, and IgAN. This study was designed to probe the effects of IgAN on intestinal microecology and metabolic phenotypes and to understand the possible underlying mechanisms. Fecal and serum samples were collected from IgAN rats. Composition of the gut microbiota and biochemical changes in the metabolites was analyzed using 16S rDNA sequencing and untargeted metabolomics. The IgAN rats exhibited renal insufficiency and increased concentration of 24-h urine protein, in addition to deposition of IgA and IgG immune complexes in the kidney tissues. There was a disturbance in the balance of gut microbiota in IgAN rats, which was remarkably associated with renal damage. Marked changes in microbial structure and function were accompanied by apparent alterations in 1,403 serum metabolites, associated with the disorder of energy, carbohydrate, and nucleotide metabolisms. Administration of Zhen Wu Tang ameliorated microbial dysbiosis and attenuated the renal damage. Besides, treatment with Zhen Wu Tang modulated the metabolic phenotype perturbation in case of gut microbiota dysbiosis in IgAN rats. In conclusion, these findings provided a comprehensive understanding of the potential relationship between the intestinal microbiota and metabolic phenotypes in rats with IgAN. Elucidation of the intestinal microbiota composition and metabolic signature alterations could identify predictive biomarkers for disease diagnosis and progression, which might contribute to providing therapeutic strategies for IgAN.

## Introduction

Immunoglobulin A nephropathy (IgAN), first proposed by Berger in 1968, is an autoimmune renal disease with multifactorial pathogenesis that has emerged as a crucial element in progression to end-stage renal disease (ESRD) (Berger and Hinglais, 1968; Lai et al., 2016). As the most common type of glomerulonephritis, the diagnosis of IgAN hinges upon an important pathological manifestation characterized by excessive accumulation and deposition of IgA immune complexes in the mesangial zone, mesangial cell proliferation, and mesangial matrix dilation (Floege and Daha, 2018; Taylor et al., 2019). In addition, urinary protein, caused by excessive deposition of IgA immune complexes, accompanied by macroscopic hematuria and hypertension, is the paramount manifestation of IgAN in the clinic. In addition, long-term studies have shown that 10–30% of IgAN patients develop kidney insufficiency and ultimately resulting in kidney failure within 20 years (Barbour et al., 2019; Jarrick et al., 2019). However, the precise pathogenesis underlying IgAN is still poorly understood. Therefore, it is of great importance to find novel and safe prevention strategies to retard the progression of IgAN.

Zhen Wu Tang (ZWT), a classic herbal prescription from China, has been widely used in the treatment of chronic kidney disease (CKD), including IgA nephropathy (Liu et al., 2018), adriamycin-induced nephropathy (Liang et al., 2019), and chronic glomerulonephritis (Wu et al., 2016). Previous studies on advantages of ZWT administration in CKD can mainly be documented into two sections: 1) ZWT has been widely used in the clinic in China for more than 1,000 years as a remedy for various kidney diseases as it displays efficacy in relieving symptoms manifested in the form of edema, dysuria, and oliguria (Liu et al., 2017b; La et al., 2018) and 2) fundamental studies indicate that ZWT possesses a range of bioactivities, including renoprotective, anti-inflammatory, diuretic, and antihyperlipidemic (Cai et al., 2010; Liang et al., 2014; Liu et al., 2019).

Interaction between the gut flora and the host is essential for maintenance of health and prevention of disease pathogenesis (Mahmoodpoor et al., 2017). The regular microbial composition, a natural defense barrier, plays pivotal roles in affecting acquisition of nutrients, modulating the immune system, and conferring metabolic capacity on the host (Claus et al., 2011). Studies reveal that ordinary intestinal flora prevent against pathogens and chronic inflammation by protecting the gut epithelial barrier structure and function (Visconti et al., 2019; Yang et al., 2019; Ho Do et al., 2020). However, changes in the intestinal mucosa epithelial barrier and gut microbes can result in the imbalance of gut microecology and further exert an influence on the surrounding apparatus (including the kidney) through multiple biological mechanisms (Schroeder and Backhed, 2016). Recent studies have recognized abnormality of intestinal flora as a new triggering element for the progression of chronic inflammatory and immune diseases (Zheng et al., 2019; Lee and Kim, 2017). Immune inflammation response has been regarded as a principal factor in the pathogenesis and progression of IgAN. Furthermore, dysfunction of the immune system results in excessive production of IgA in the circulation, further stimulating the gastrointestinal mucosa, which plays a central role in the pathogenesis of IgAN. Notably, IgAN causes dysbiosis of gut microbiota and intestinal epithelial barrier disruption leading to overproduction of various uremic toxins, which accelerate the progression of IgAN to ESRD. Increasing number of clinical observations, either directly or indirectly, has suggested that the gut–kidney axis exerts a major effect on the pathogenesis and progression of IgAN (Cao et al., 2018; Hu et al., 2020). Pioneering research has indicated that IgAN patients display aberrant gut histopathological patterns and symptoms of gut inflammation (Wang et al., 2004; Chemouny et al., 2019). In addition, based on the theory of the gut–kidney axis, regulation of the intestinal microecology has been found to be beneficial in preventing and controlling the development of kidney diseases. Therefore, it is of great importance to explore the microbiota that plays a critical role in the development and evolution of a disease. Precise manipulation of the microbiota may serve as a promising therapeutic objective in the future.

The gut microbiota plays a pivotal part in the metabolic activities of the host, including digestion of complex polysaccharides, modulation of the immune system, synthesis of certain endogenous vitamins and amino acids, and metabolism of bile acids (Nicholson et al., 2012; Oliphant and Allen-Vercoe, 2019). Dysbiosis of the composition of the gut microbiota can profoundly affect the levels of derived metabolites, including short-chain fatty acids (SCFAs), uremic toxins p-cresol sulfate and indoxyl sulfate, displays either pro-inflammatory or anti-inflammatory activities, and exerts critical roles in the evolution of AKI and CKD (Tang et al., 2015; Yacoub and Wyatt, 2017). A few recent studies have demonstrated that an increase in intestinal permeability facilitates the translocation of endotoxins into systemic circulation, leading to the activation of innate immunity systemic inflammation, which plays a central role in CKD (Xu et al., 2017; Feng et al., 2019). Available evidence illustrated the bidirectional effects between gut flora and hosts with CKD. SCFAs, as metabolites of beneficial bacteria, can prevent ischemia reperfusion kidney injury by diminishing the inflammatory response and promoting apoptosis (Felizardo et al., 2019). However, other potentially toxic metabolites from bacteria, including p-cresol sulfate and indoxyl sulfate, can promote the release of pro-inflammatory cytokines, and thereby, accelerate kidney injury. Hitherto, few research studies have been performed to explore the causal connection between intestinal microbiota and related metabolites in IgAN pathogenesis.

Above all, the aims of the present study were designed to dissect the direct relationship between gut microbiome and metabolome as well as their effects on IgAN rats, using a combination of 16S rDNA sequencing and untargeted metabolomics analysis. Due to the complicated pathogenesis of IgAN and the inconclusive relationship between gut microbiota, serum metabolites, and IgAN, it is rather necessary to establish a relevant biological association between the gut–kidney axis and IgAN using the gut microbiomics and metabolomics in future studies. Emerging proof from clinical, genetic, and immunological research supports the finding that intestinal flora dysbiosis plays an important role in the development and evolution of kidney disease (Wang et al., 2020). Moreover, preliminary studies have illustrated that immune-sensing inflammation is regarded as a principal factor in the development of IgAN prior to ESRD (Coppo, 2017; Xie et al., 2018). Based on the findings on the potential effect of gut flora on modulating immunity and suppressing inflammation, exploring gut microorganisms and related metabolic biomarkers is of great significance for the early diagnosis and treatment of IgAN.

## Materials and Methods

### Experimental Animals

**FIGURE 1:**
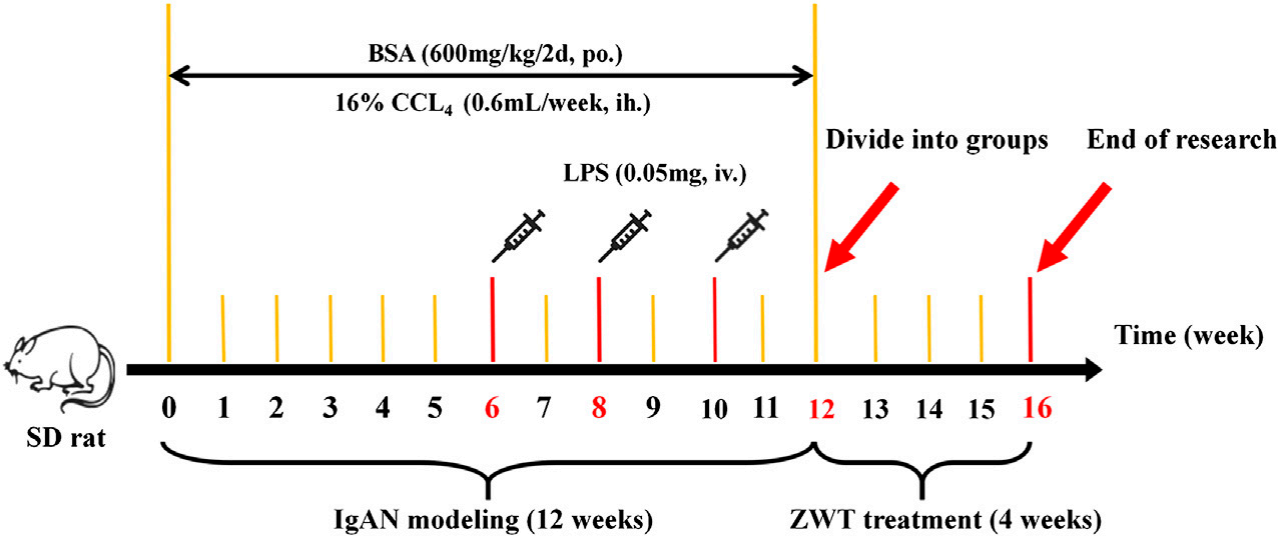
Experimental timeline of IgAN modeling and ZWT treatment.

A total of 24 healthy male special pathogen-free (SPF) Sprague–Dawley rats (weighing between 180 and 220 g) obtained from the Medical Science Experimental Animal Center of Guangdong Province in China (license no SCXK 2018–0002) were used in this study. The animals were housed under SPF conditions at 25 ± 2 C and 65% humidity, with a 12 h/12 h light/dark cycle. The rats were given free access to standard laboratory rat chow and tap water. After acclimation for 1 week, eight rats were grouped into the control group, while the rest of rats were administered BSA, LPS, and CCl_4_ to establish the IgAN model. In detail, in the first week, every model rat was orally administered 600 mg/kg BSA every other day for 12 weeks. Next, the rats were injected with 0.05 mg LPS through the tail vein on the first day of the sixth, eighth, and 10th weeks. The rats were then subcutaneously injected with 0.1 ml CCl_4_ dissolved in 0.6 ml castor oil every week for 12 weeks. Following that, the model rats, whose 24-h urinary protein levels were higher than those of the normal rats, were randomly divided into two groups (eight rats per group)—IgAN model group and ZWT treatment group (16.8 g/kg). Rats in the control and model groups were administered 10 ml/kg/d saline using the oral gavage method (Bai et al., 2019b; Liu et al., 2017a; Yang et al., 2018). Drug treatments were performed by administering the corresponding drugs once daily for 4 weeks (Liu et al., 2018; Li et al., 2020). The procedures of the animal experiment were approved by the Animal Ethics Committee of Guangzhou University of Chinese Medicine and performed according to the European Community Guidelines and the regulations of the National Institute of Health of USA. All efforts were made to minimize the suffering of the animals (Figure 1).

### Preparation of Zhen Wu Tang

ZWT, a traditional Chinese formula, which consists of five herbs (as shown in Table 1 **)**, was purchased from Kangmei Pharmaceuticals Co. Ltd. (lot nos. 190305041, 190250291, 190103501, and 190103501). Shengjiang (*Zingiber officinale Roscoe*) was purchased from Maisi supermarket in Guangzhou Higher Education Mega Center. As previously described, to prepare the clinical dose of ZWT, aqueous extracts of the different herbs were mixed in the ratios given in Table 1. Briefly, ZWT was boiled for 2 h after being steeped eight times in distilled water for 1 h. After repetition of the same extraction conditions three times, the combined filtrates were used to obtain a final aqueous extract with a concentration of 1.68 g raw materials per milliliter. The extract was stored at 4°C before use. The characterization of ZWT using HPLC has been described in a previous study (Liu et al., 2019; Li et al., 2020).

**TABLE 1:** Composition of ZWT.

Scientific name	Chinese name	Ratio
*Aconitum carmichaelii* Debeaux	Fuzi	3
*Poria cocos (Schw.)* Wolf	Fuling	3
*Atractylodes macrocephala* Koidz	Baizhu	2
*Paeonia lactiflora* Pall	Baishao	3
*Zingiber officinale* Roscoe	Shengjiang	3

### Biochemical Analysis of Urine and Blood Samples

After the last drug administration, 24-h urine samples were collected from the animals to measure the levels of urinary protein using Super-Bradford Protein Assay Kits (CWBIO, Beijing, China). During the urine collection period, all the rats were forbidden from food but given free access to water. Subsequently, the rats were weighed and sacrificed by administering an intraperitoneal injection with 35 mg/kg pentobarbital sodium to collect blood samples and renal tissues. Blood collected from the abdominal aorta was centrifuged at 3,000 rpm for 10 min at 4°C to acquire serum for subsequent biochemical analysis. In addition, the ratio of absolute kidney and spleen weight to body weight (kidney and spleen index) was measured. The levels of blood urea nitrogen (BUN) and creatinine (CRE) in the serum were detected using kits for BUN and CRE measurements, respectively, according to the manufacturer's instructions (Nanjing Jiancheng Bioengineering Institute, Nanjing, China).

### Histological Analysis

Renal and colon damage were assessed using periodic acid–Schiff (PAS) staining and hematoxylin–eosin (HE) staining, as described previously. Immunofluorescence procedures for IgA and complement 3 in the renal tissue were carried out as described previously. The stained slides were histopathologically examined and photographed under a light microscope (Olympus BX53, Shanghai, China).

### High Throughput Sequencing of Fecal DNA

The feces of each animal were collected in a sterile EP tube and stored at −80°C for DNA extraction. Microbial genomic DNA was extracted from stool samples using the E.Z.N.A^®^ Soil DNA Mini Kit (R6825, Omega Bio-Tek, Norcross, GA, United States), in accordance with the manufacturer’s protocols. The concentrations and purity of the resultant DNA samples were assessed using a NanoDrop™ system (NanoDrop, United States), following which the DNA was stored at −80°C for further tests.

The 16S rDNA gene was amplified using PCR with primers 16S-F (5′-CCTACGGGNGGCWGCAG-3′) and 16S-R (5′-GGACTACHVGGGTATCTAAT-3′), which target the V3–V4 region of the bacterial 16S rDNA gene. PCR reactions were performed in triplicate with Phusion^®^ High-Fidelity PCR Master Mix (New England Biolabs) using template DNA. PCR products were purified using the AxyPrep™ DNA Gel Extraction Kit (Axygen Biosciences, Union City, CA, United States), according to the manufacturer’s instructions and quantified using ABI StepOnePlus™ Real-Time PCR System (Life Technologies, Foster City, CA, United States). The purified PCR products were pooled in an equimolar ratio and paired-end sequenced (2 × 250) on an Illumina HiSeq 2,500 Sequencing System (Illumina, San Diego, CA, United States) according to standard protocols (Caporaso et al., 2012).

### Bioinformatics Analysis of 16S rDNA Gene Sequences

Raw tags were filtered to delete adapters or low-quality tags and quality filtered using FASTP (https://github.com/OpenGene/fastp) with the following criteria: i) tags containing more than 10% of unknown nucleotides (N) were removed and ii) tags containing less than 60% of bases with quality (Q-value) > 20 were removed. Paired-end clean tags were merged as raw tags using FLASH (version 1.2.11) with a minimum overlap of 10 bp and mismatch error rates of 2%. Noisy sequences of raw reads were filtered using QIIME pipeline (version 1.9.1) to obtain high-quality clean tags (Caporaso et al., 2010). The clean tags were then merged using the reference database (http://drive5.com/uchime/uchimedownload.html) to perform reference-based chimera checking with the UCHIME algorithm. The effective tags were clustered into a 97% similarity cutoff using the UPARSE pipeline (Edgar, 2013). Each 16S rDNA gene sequence was analyzed and classified into organisms on the basis of a naive Bayesian model using RDP classifier (version 2.2), based on the SILVA database (https://www.arb-silva.de), with confidence threshold values between 0.8 and 1. The sequence data reported in the study have been archived in the NCBI Sequence Read Archive (http://www.ncbi.nlm.nih.gov/sra) with the accession number SRP284194.

### Sample Extraction and LC-MS Analysis for Metabolomics

Metabolites in serum were analyzed using untargeted metabolomics LC-MS. 100-μL serum sample was taken and placed into an EP tube. After the addition of 300 μL methanol (containing 1 μg/ml internal standard), the samples were vortexed for 30 s, sonicated for 10 min (incubated in ice water), and incubated for 1 h at −40°C to precipitate the proteins. The sample was then centrifuged at 10,000 rpm for 15 min at 4 C. The resulting supernatant was transferred to a fresh glass vial for analysis.

LC-MS/MS analysis was performed using a UHPLC system (1290, Agilent Technologies) with a UPLC HSS T3 column (2.1 mm × 100 mm, 1.7 μm) coupled to a Q Exactive™ mass spectrometer (Orbitrap MS, Thermo Fisher Scientific). The mobile phase A consisted of 0.1% formic acid in water for positive mode and 5 mmol/L ammonium acetate in water for negative mode, while the mobile phase B was acetonitrile used as an elution gradient. The proportion of acetonitrile was varied from 1% to 99% in 12 min (0–1.0 min, 1% B; 1.0–8.0 min, 1–99% B; 8.0–10.0 min, 99% B; 10.0–10.1 min, 99–1%; 10.1–12 min, 1% B) at a flow rate of 0.5 ml min^−1^. The injection volume of each sample was set at 2 μL. The MS parameters were set as follows: interface of negative electrospray ionization: ion spray voltage floating 3,800 V or −3,100 V in positive or negative modes, respectively; sheath gas flow rate at 45 Arb; aux gas flow rate at 15 Arb; capillary temperature 400°C; full MS resolution at 70,000; MS/MS resolution at 17,500; collision energy at 20/40/60 eV in NCE mode; spray voltage at 4.0 kV (positive) or −3.6 kV (negative). R package XCMS (version 3.2) was employed for the analyses (Want et al., 2010; Xu et al., 2017).

### Data Processing and Annotation

MS raw data files were converted into the mzML format using ProteoWizard and processed using R package XCMS (version 3.2), by carrying out retention time alignment, peak detection, and peak matching. The data were then filtered using the following criterion: sample numbers containing a metabolite were less than 50% of all sample numbers in a group. Subsequently, each sample was normalized to an internal standard. The processing results generated a data matrix consisting of the retention time, mass-to-charge ratio values, and peak intensity. OSI-SMMS (version 1.0, Dalian Chem Data Solution Information Technology Co. Ltd.) was used for peak annotation after data processing with an in-house MS/MS database.

### Statistical Analysis

Statistical analyses were performed using SPSS 26.0 software (Chicago, IL, United States) and GraphPad Prism software 7.0. PCA, PLS-DA, and OPLS-DA were performed using SIMCA-P software to cluster the sample plots across groups. Alpha-diversity analysis, including richness, Chao1, Simpson, and Shannon diversity indices were calculated using Wilcoxon rank-sum test in QIIME. Beta-diversity analysis was performed using Muscle (version 3.8.31) (http://www.drive5.com/muscle/). Functional prediction of 16S rDNA gene sequence was carried out using PICRUSt (version 1.0). KEGG (http://www.kegg.com/) database was used for identification of potential metabolite markers; those with variable importance in the projection (VIP) value >1.0 and *p* value < 0.05 were included. Association between serum metabolite intensities and gut flora was tested using Pearson’s rank correlation. The metabolism network was established using Cytoscape (version 3.7.0). All the data have been presented as mean ± standard deviation. Significance level of differences between two groups was analyzed using Student’s unpaired t-test, while multiple comparisons were made using one-way ANOVA, followed by Duncan's *post hoc* test. *p* < 0.05 was considered statistically significant.

## Results

### Sequencing Data Quality

**FIGURE 2:**
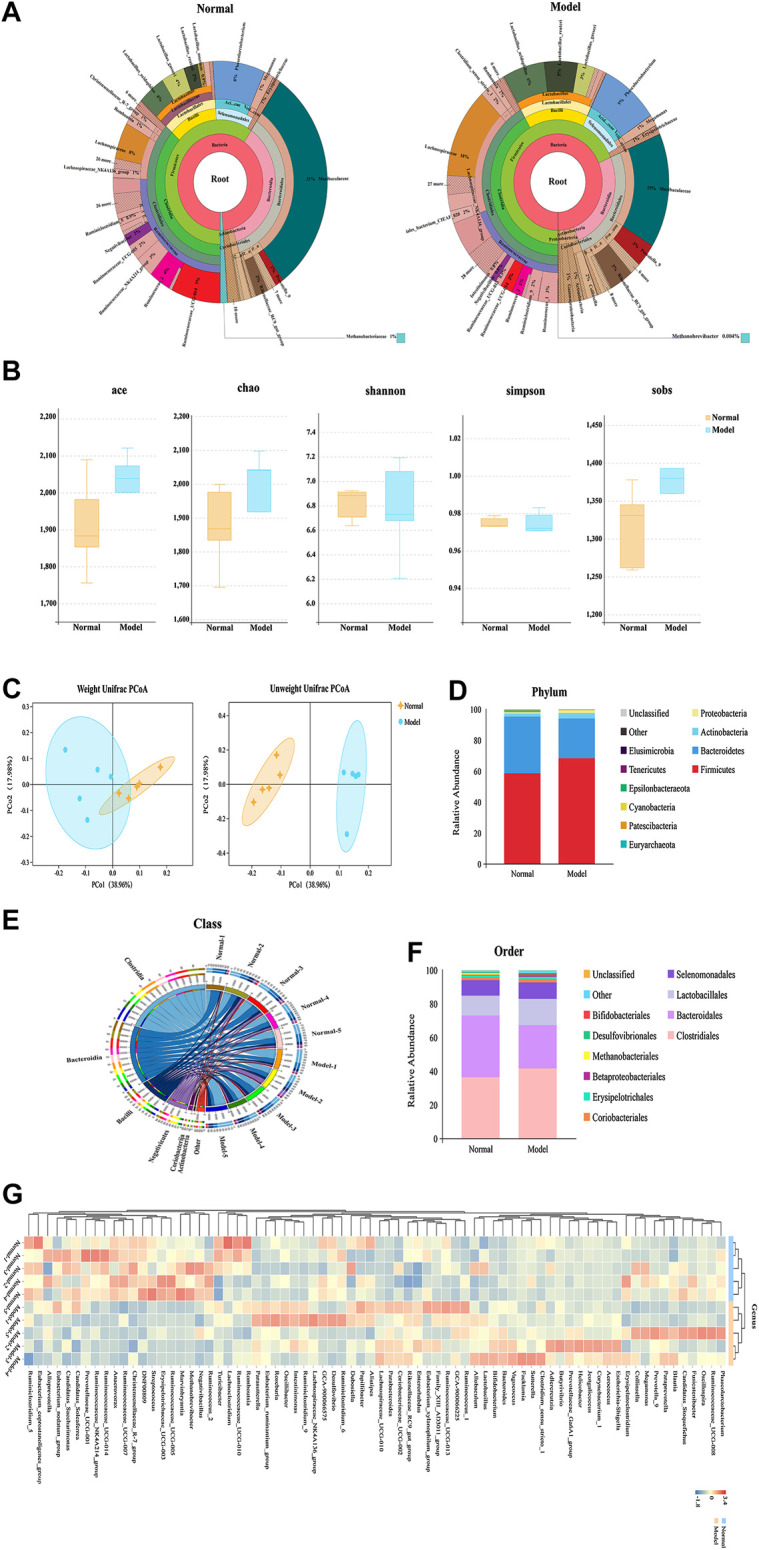
Comparative analysis of fecal microbial diversity and composition in IgAN rats. **(A)** Cladogram of differentially abundant taxa between normal and model. **(B)** The alpha-diversity indices (Ace, Chao1, Shannon, Simpson, and Sobs) of the normal and IgAN model groups were calculated based on OTU levels. **(C)** Principal coordinate plot showing the beta diversity between the normal and model rats, based on unweighted and weight ed UniFrac analyses. *p* value indicates differential clustering, as evaluated using the ADONIS test. **(D)** Component proportion of microbiota between the normal and model groups from fecal 16S rDNA sequencing data at the phylum level (top 10). **(E)** Stacking diagram of species distribution between normal and model groups at the order level (top 10), as revealed using 16S rDNA sequencing. **(F)** Circos showing the microbial composition of each sample from the normal and model groups at the class level (top 10). **(G)** Heatmap displaying the microbial composition of each sample from the normal and model groups at the genus level.

In our microbiome study, 1,416,455 raw tags of microbial 16S rDNA were acquired from fecal samples using Illumina sequencing. Illumina MiSeq sequencing analysis was applied to identify the composition of gut flora in the fecal samples of rats. 1,309,828 effective tags were acquired after filtering the data quality, with an average number of 87,322 tags per sample (range: 82,730 to 93,921) (Supplementary Table S1). These sequences were assigned to 1,620 operational taxonomic units (OTUs) based on 97% sequence resemblance. According to the taxonomic assignment, a total of 1,422 OTUs were identified from the normal and IgAN model groups with a median read count of 1,333.8 OTUs (range: 1,180–1,450) per sample (Supplementary Table S2). OTUs with abundance greater than 1% were selected and used to construct a species classification diagram (Figure 2A).

### Changes in Bacterial Diversity in Fecal Microbiota of Immunoglobulin A Nephropathy Rats

Alpha-diversity indices including Chao1, Shannon, and Simpson were calculated to evaluate differences in the ecological diversity of microbial communities between the normal and IgAN model groups. According to the Wilcoxon rank-sum test, there were no significant differences in the Chao1 (1874.20 ± 122.02 vs. 1948.38 ± 182.61), Ace (1912.48 ± 127.48 vs. 1984.97 ± 169.50), Sobs (1315.00 ± 52.61 vs. 1352.60 ± 102.12), Shannon (6.81 ± 0.13 vs. 6.78 ± 0.39), and Simpson (0.9751 ± 0.0027 vs. 0.9717 ± 0.0113) indices between the normal and IgAN model groups, respectively (Figure 2B). Both unweighted (ADONIS) (*p* = 0.005) and weighted (ADONIS) (*p* = 0.012) UniFrac analyses of the PCA plots exhibited marked discrepancies between the normal and IgAN model groups in terms of beta diversity (Figure 2C). Consequently, the above results demonstrated that IgAN could have a significant influence on the diversity and composition of the gut microbiome.

### Comparative Analysis of Fecal Microbial Composition Associated with Immunoglobulin A Nephropathy

According to the data from the phylum to genus level, composition of the gut microbiome varied greatly between the groups; this variation could be an influence of IgAN. Based on the taxonomy stack distributions at the phyla level, all of the observed sequences were aligned and classified into 19 phyla, 28 classes, 48 orders, 73 families, 184 genera, and 94 species (Supplementary Table S3). The relative proportions of dominant taxa were evaluated by microbial taxon assignment of the normal and IgAN model groups. Firmicutes (58.71% vs. 68.43%) and Bacteroidetes (36.64% vs. 25.72%), which are the representative intestinal flora structures in rats, were the dominant gut microbiota in both the normal and IgAN rats, respectively. Other phyla such as Proteobacteria, Euryarchaeota, and Cyanobacteria were found at low levels (less than 4%) (Figure 2D). According to the Wilcoxon rank-sum test, only the relative abundance of Cyanobacteria and Euryarchaeota was obviously reduced (*p* < 0.05) in the IgAN model group, as compared to the normal group. At the class level profiles, the relative abundance levels of Clostridia, Bacteroidia, Bacilli, and Negativicutes were predominant in both the groups, with no remarkable differences between the two groups (Figure 2E). At the order level, there was a notable increase in the abundance of Betaproteobacteriales, while Methanobacteriales were significantly reduced in the IgAN model group (Figure 2F). At the genus level, an average of 40.08 and 33.27% of the sequences per sample could not be assigned to specific genera in the normal and IgAN groups, respectively. The remaining sequences were assigned to 184 genera, of which *Lactobacillus*, Phascolarctobacterium, and Ruminococcaceae_UCG-014 were abundant in both the groups (Figure 2G).

### Identification of Immunoglobulin A Nephropathy-Specific Fecal Microbial Communities

**FIGURE 3:**
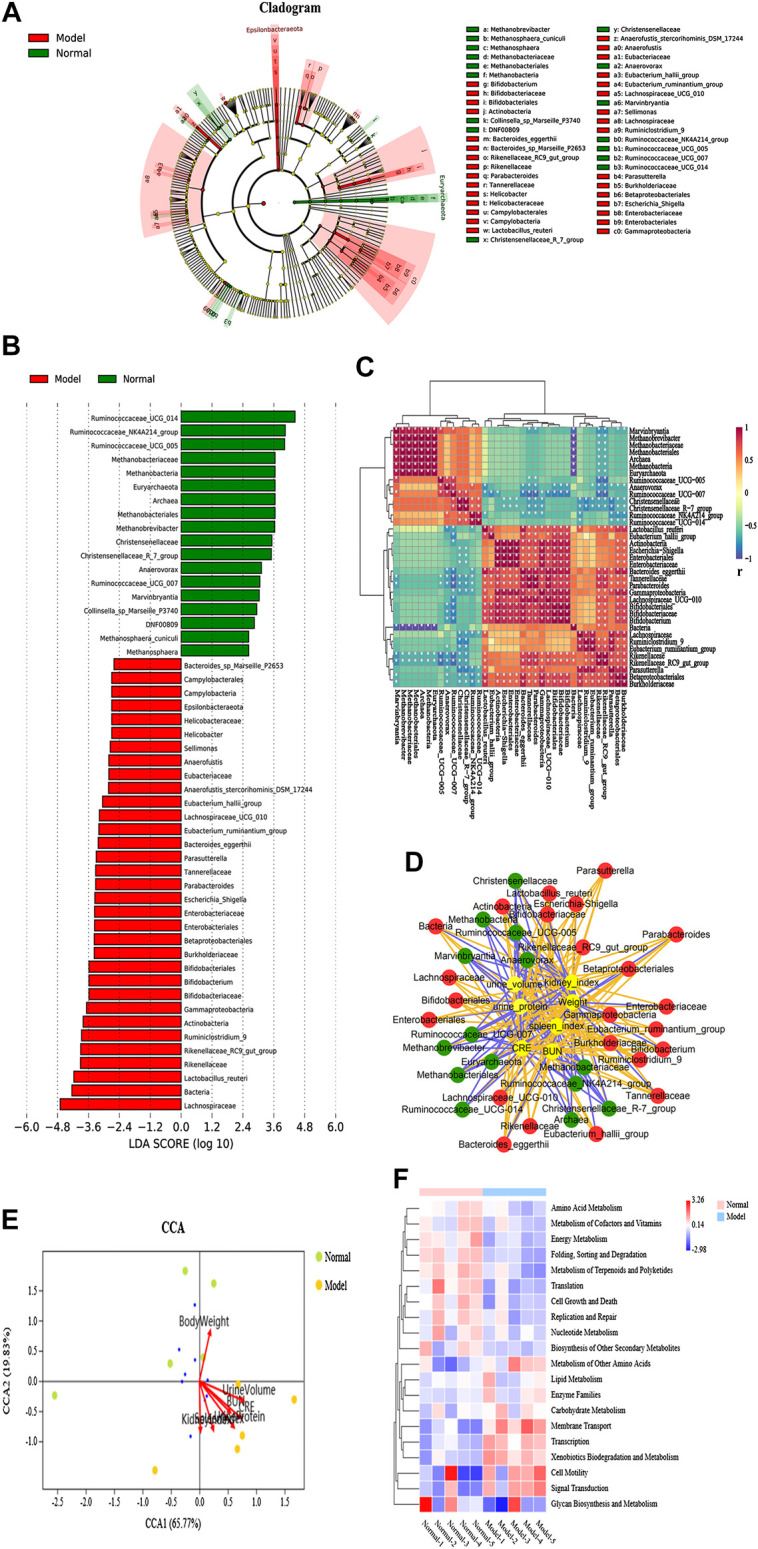
Specific fecal microbial communities and potential functional pathways in IgAN rats. **(A)** Cladogram displaying the specific bacterial taxa between the normal and model groups (phylum to genus level). **(B)** LEfSe analysis of the specific bacterial taxa of the normal and model groups (LDA score > 3.0, Wilcoxon rank-sum test *p* < 0.05, n = 5). **(C)** CCA analysis of biochemical parameters and microbial communities at the phylum level. The direction and length of the red lines indicates the contribution of the corresponding parameter to the microbial communities. **(D)** Interaction network diagram of the biochemical parameter and specific microbes, based on Pearson’s correlation analysis. The orange line represents a positive correlation, while the blue line represents a negative correlation. **(E)** Heatmap showing Pearson’s analysis of discriminant bacterial species in the normal and model groups. Significant differences were determined using Wilcoxon rank-sum test, *p < 0.05. **(F)** KEGG functional prediction analysis of altered microbial communities associated with IgAN rats.

LEfSe analysis was applied to distinguish the predominant gut microbiota that could induce renal dysfunctions and change metabolic pathways. The specific bacterial taxa in the IgAN feces have been represented using a cladogram, while the column graphs represent the linear discriminant analysis scores. As shown in Figures 3A and B, the IgAN rats contained higher enrichment of *Lactobacillus*_*reuteri*, Rikenellaceae_RC9_gut_group, Ruminiclostridium_9, Actinobacteria, and Bifidobacterium, while the normal rats primarily contained higher enrichment of Ruminococcaceae_UCG_014, Ruminococcaceae_NK4A214_group, Ruminococcaceae_UCG_005, and Methanobrevibacter as well as Christensenellaceae_R_7_group, reflecting regular kidney function (LDA score > 3.0 with *p* < 0.05). Thus, this finding identified the differentially distributed microbial communities as biomarker candidates for IgAN, which showed great promise for precision medicine. Therefore, novel insights into the disturbance of gut microbiota in the condition of IgAN are helpful to identify potential therapeutic targets and develop novel therapeutic strategies for preventing or attenuating the disease progression.

### Association of the Microbial Dysbiosis and Dysregulation of Biochemical Parameters in Immunoglobulin A Nephropathy

The heatmap in Figure 3C illustrated that 37 taxa were obviously distinct between the IgAN rats and the normal rats. In addition, Pearson’s correlation analysis further confirmed the critical taxa that were strongly correlated with IgAN rats. Pearson’s correlation analysis was utilized to identify bacteria related to changes in biochemical parameters in IgAN rats. The result indicated that CRE and urine protein was deeply implicated in the changes in intestinal flora in IgAN rats. Notably, the result revealed that plasma BUN and CRE levels, 24-h protein urine, weight, spleen index, kidney index, and urine volume were positively or negatively associated with the richness of several genera (Figure 3D). In order to further clarify the effect of biochemical parameters on gut flora, canonical correspondence analysis (CCA) was carried out at the OTU level. Among others, the first two components (CCA1 and CCA2) further illustrated the contribution of biochemical parameters to change bacterial structure and composition (Figure 3E). In summary, these findings indicated that there is a potential link between gut microbiota dysbiosis and kidney insufficiency. However, further investigations need to be carried out to understand this relationship to a greater extent.

### Key Metabolic Pathways Associated with Differential Fecal Microflora in Immunoglobulin A Nephropathy Rats

PICRUSt analysis was carried out for functional profiling of the gut microbiota from all samples using the 16S rDNA data. Biological metabolic pathways can be classified into seven categories, namely, metabolism, genetic information processing, environmental information processing, cellular processes, organismal systems, human diseases, and drug development. The data in this study suggested that vital alterations in the gut microbial gene functions were associated with metabolism. 3,196 KEGG Orthology (KO) terms were determined using PICRUSt analysis, which were found to be associated with 21 significantly altered metabolic pathways in IgAN (Supplementary Table S4), such as adipocytokine signaling pathway, D-arginine and D-ornithine metabolism, flavone and flavonol biosynthesis, biotin metabolism, riboflavin metabolism, and caprolactam degradation. Interestingly, xenobiotics biodegradation and metabolism, alpha-linolenic acid metabolism, ether lipid metabolism, and glutathione metabolism were found to be upregulated, while cofactor and vitamin metabolism, amino acid metabolism, energy metabolism, citrate cycle (TCA cycle), and D-glutamine and D-glutamate metabolism were found to be downregulated in the IgAN rats. Consequently, alterations in bacterial communities in IgAN might potentially trigger alterations in a variety of microbial metabolic pathways, ultimately affecting the disease outcome (Figure 3F).

### Altered Serum Metabolic Profiling in Immunoglobulin A Nephropathy Rats

**FIGURE 4:**
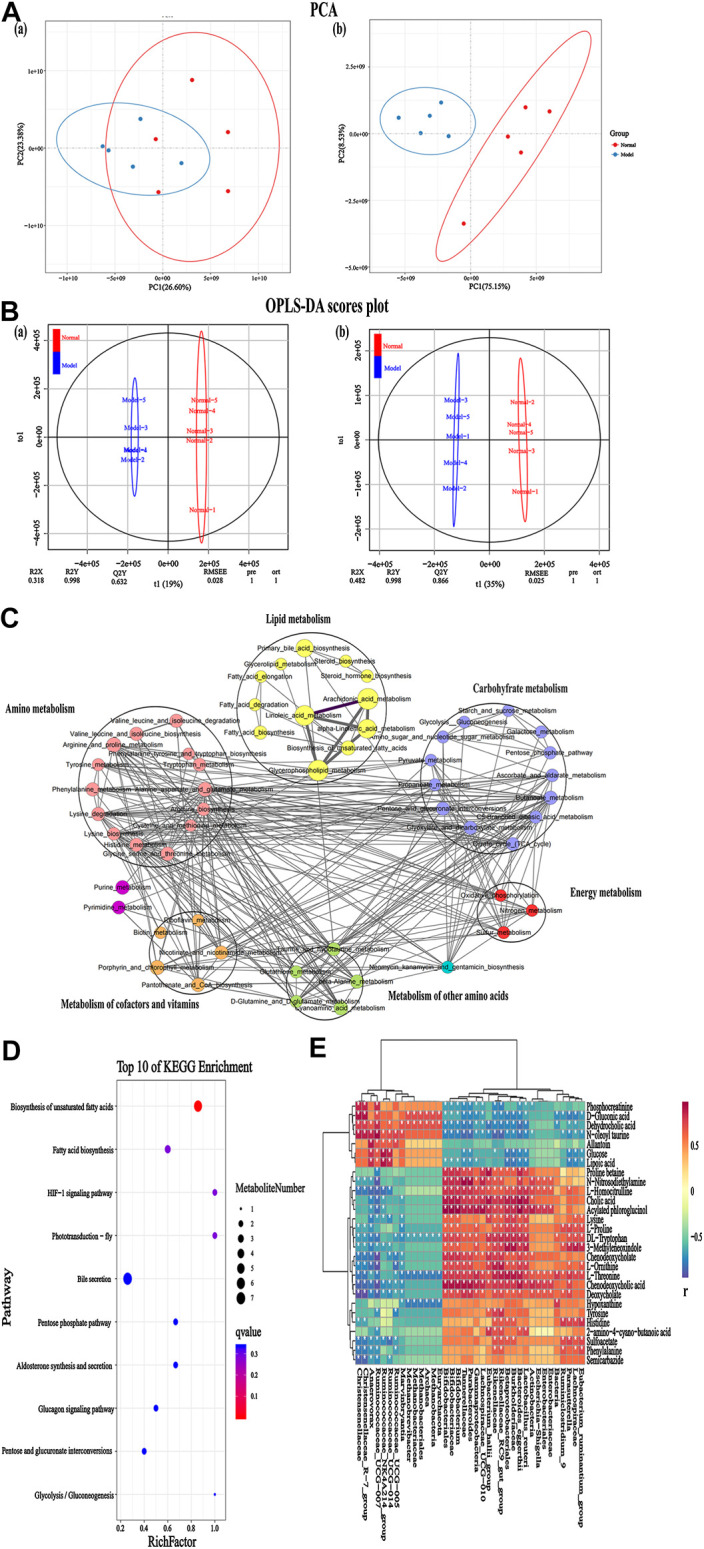
Serum metabolomics for identification of metabolites and metabolic pathways in the IgAN rats. **(A)** PCA plots [positive **(A)** and negative **(B)** ion mode] revealed that the overall metabolite profiles differed between the normal and model rats (n = 5). **(B)** OPLS-DA plots [positive **(A)** and negative **(B)** ion mode] with the scores of the first two principal components from the normal and model rats (n = 5). **(C)** Integrated metabolite pathway-based network analysis of serum metabolites using Cytoscape software, based on KEGG database. **(D)** Top 10 altered metabolites between the normal and model groups, based on KEGG pathway enrichment analysis and statistical significance. **(E)** Heatmap representing the correlation values between discriminant bacterial species and differential serum metabolites in the normal and model groups. Asterisks represent the significance level of the corresponding correlation coefficient, *p < 0.05.

Serum metabolic profiling using PCA score plots [positive (a) and negative (b) ion mode] revealed that there was a significant separation between the normal group and the IgAN model group (Figure 4A). In addition, further OPLS-DA model analysis also confirmed that the two groups were clearly separated, indicating that there were significant alterations in the physiology and body metabolic status of IgAN rats (Figure 4B). According to the OPLS-DA scores plot, R2X and R2Y were employed to analyze the interpretation rate of the matrix, while Q2Y represented the predictive ability of the model. The performance statistics were as follows: R2X = 0.318 or 0.482, R2Y = 0.998, and predictive parameter Q2Y = 0.632 or 0.866, which suggested that the IgAN model was credible. S-plots and VIPs of OPLS-DA were employed to seek out critical markers in the IgAN model. 787 (positive ion mode) and 616 (negative ion mode) significantly altered metabolic biomarkers were selected (VIP > 1, *p* < 0.05) using multivariate statistical analysis of serum samples from IgAN rats; the same have been presented in the heatmap (Supplementary Table S5). These biomarkers of serum metabolites were mainly involved in lipid metabolism, signal transduction, carbohydrate metabolism, amino acid metabolism, and biosynthesis of other secondary metabolites (Figure 4C). The metabolic pathways enriched by the metabolites were consistent with the potential metabolic function predicted by the above intestinal flora, which indicated that intestinal flora could affect the development of IgAN by altering metabolites. The data of pathway analysis reflected that there were ten principal altered pathways of serum metabolites in IgAN rats, which included: 1) biosynthesis of unsaturated fatty acids, 2) fatty acid biosynthesis, 3) HIF-1 signaling pathway, 4) phototransduction-fly, 5) bile secretion, 6) pentose phosphate pathway, 7) aldosterone synthesis and secretion, 8) glucagon signaling pathway, 9) pentose and glucuronate interconversions, and 10) glycolysis/gluconeogenesis (Figure 4D).

### Correlation Analysis for Immunoglobulin A Nephropathy–Induced Fecal Microbial Dysbiosis and Dysregulation of Metabolites

A link between the microbiome community structure and metabolic signatures could reflect a predictive profile for IgAN progression. To analyze such associations and explore the underlying potential mechanisms, Pearson’s correlation coefficient was applied on the above fecal microbiome and metabolomics data. The results revealed the specific metabolites that were positively or negatively associated with the richness of several genera, indicating that the differentially enriched metabolites in IgAN rats were possibly responsible for the imbalance in intestinal flora (Figure 4E). This finding provided novel insight into the investigation of gut microbiome–metabolome associations for new target discovery.

### Effects of Zhen Wu Tang on Treating Immunoglobulin A Nephropathy Rats

#### Effects of Zhen Wu Tang Treatment on Fecal Microbiota Dysbiosis

**FIGURE 5:**
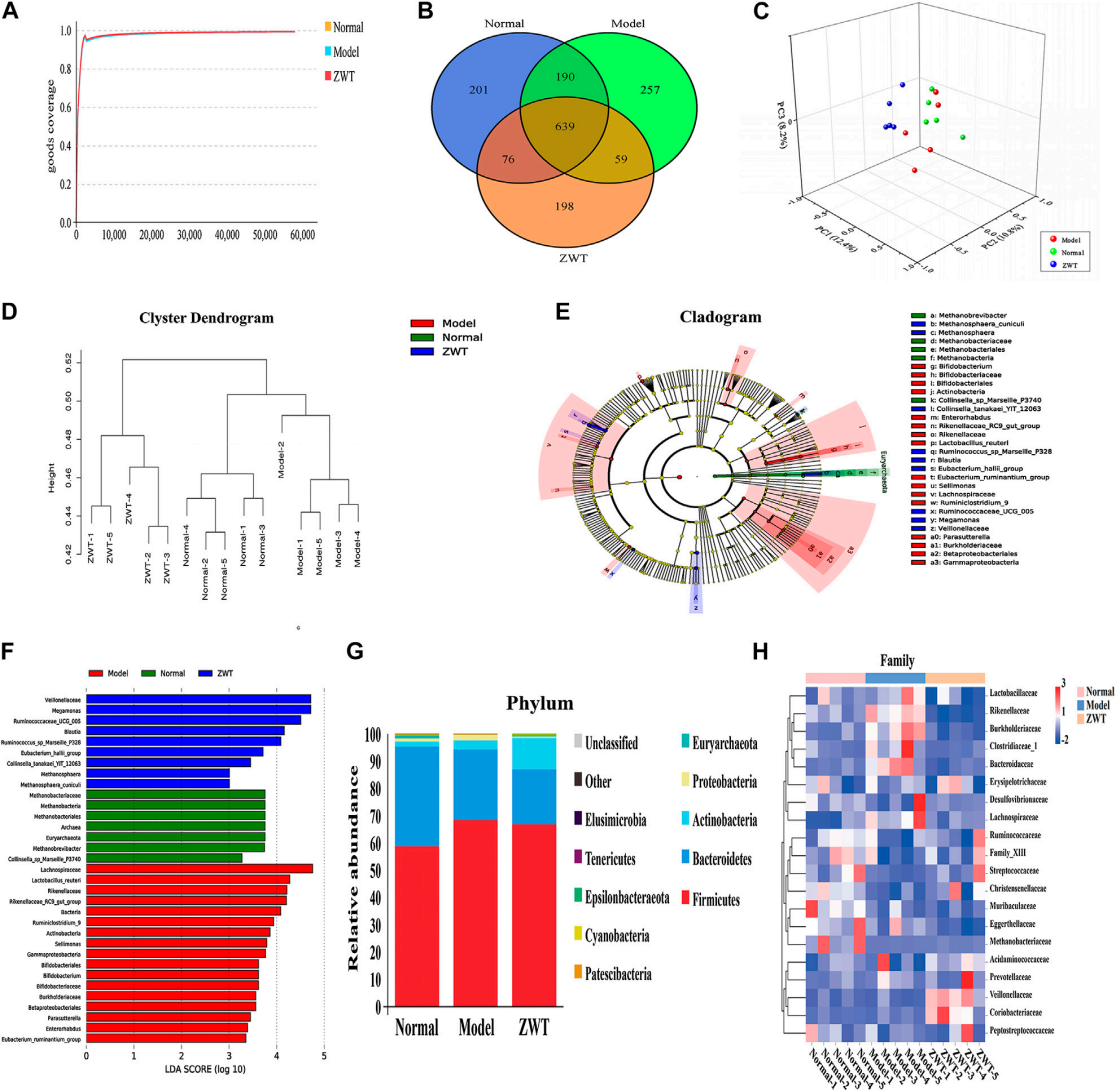
Effect of ZWT on gut microbiota of IgAN rats. **(A)** Shannon rarefaction curves of normal, model, and ZWT groups. **(B)** Hierarchical clustering analysis of UPGMA in three groups based on unweighted UniFrac analysis. **(C)** 3D-PCA was executed in the three groups, based on OTU levels. **(D)** Venn diagram showing observed OTU numbers among the three groups. Orange symbols indicate ZWT group; blue symbols, normal group; and green symbols, model group. **(E)** Phylogenetic tree in cladogram format displaying discriminatory bacterial taxa among the three groups, based on LEfSe analysis (phylum to genus level). **(F)** The difference in abundance levels of bacterial taxa among the three groups (LDA score > 3.0, Wilcoxon rank-sum test *p* < 0.05). **(G)** Stacking diagram of species distribution between normal, model, and ZWT groups at the phylum level from fecal 16S rDNA sequencing data (top 10). **(H)** Heatmap showing the component proportion of microbiota of each sample from the normal, model and ZWT groups at the family level (top 20).

To illustrate whether intestinal flora influence IgAN, we treated IgAN rats with ZWT, a classic herbal prescription with treatment effects on CKD that has been in clinical practice since centuries (Liang et al., 2019). Figure 5A demonstrated that the Shannon rarefaction curves for each group approached a saturation plateau, suggesting the adequacy of sequence coverage for each sample. Venn diagrams were made to detect differences in OTUs between the different groups, which summarized that the general numbers of OTUs were 1,290, 1,146, and 964 for the normal, model, and ZWT treatment groups, respectively (Figure 5B). In terms of specific flora, 257 OTUs were observed only in the model group, while 198 OTUs were observed only in the ZWT-treated group. In addition, there were 639 overlapping OTUs that were shared by all the three groups (Supplementary Table S6). 3D-PCA and UPGMA were used to present different taxonomic compositions in the three groups, showing satisfactory clusters among them (Figures 5C,D). Indeed, the result exhibited that the gut microbiota from IgAN rats treated with ZWT tended to approach that from normal rats, suggesting a meaningful ZWT-mediated modulation on the bacterial community. LEfSe analysis showed that there was a significant change in the specific and predominant bacteria in response to ZWT treatment. *Lactobacillus*_*reuteri*, Rikenellaceae, and Bifidobacterium were predominant in the model rats; Eubacterium_hallii_group, *Megamonas*, and Blautiawere were specific for the ZWT-treated model rats; and the normal rats were characterized by Methanobrevibacter and Collinsella_sp_Marseille_P3740. The results indicated that gut flora homeostasis could be perturbed by IgAN, while ZWT could ameliorate the gut microbiota dysbiosis (Figures 5E,F). Intestinal flora analysis revealed that the model groups exhibited different degrees of disorder at the phylum level, as compared to the normal group. The levels of Cyanobacteria and Proteobacteria could be restored to the control level upon ZWT treatment (Figure 5G). Additionally, we observed that ZWT administration increased the relative abundance of microbial species at the family level, including *Peptostreptococcaceae*, *Methanobacteriaceae*, *Christensenellaceae*, *Streptococcaceae*, and Family_XIII. Moreover, the model group had higher abundance of *Lachnospiraceae*, *Lactobacillaceae*, *Erysipelotrichaceae*, and *Bacteroidaceae* relative to the normal group; these levels were found to be reduced upon ZWT intervention (Figure 5H). These data suggested that ZWT exerted a protective role in IgAN rats through regulation of the microbial structure.

#### Effect of Zhen Wu Tang Treatment on Serum Metabolic Profiles

**FIGURE 6:**
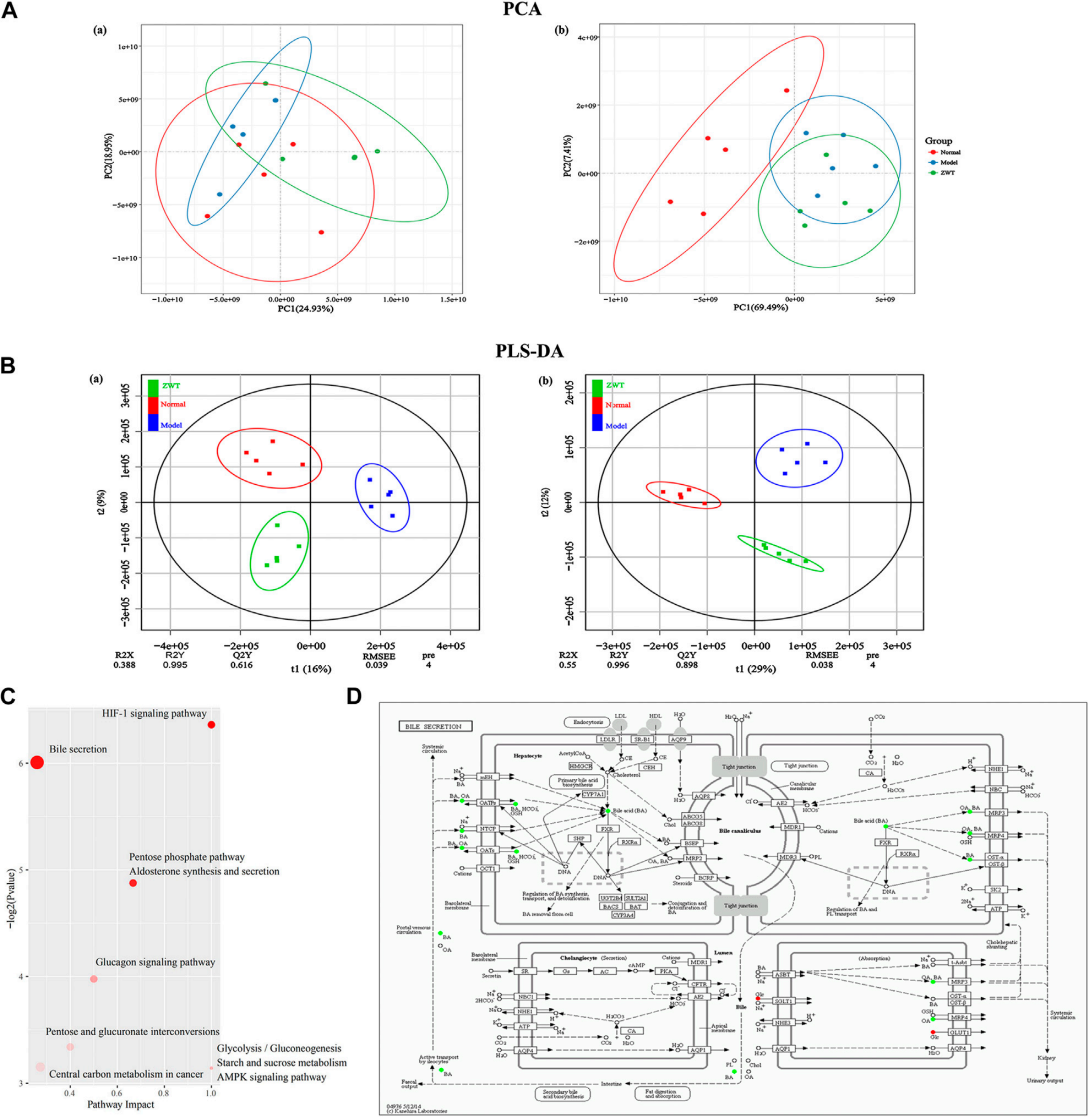
Change in metabolic profiles of IgAN rats upon ZWT treatment. **(A)** PCA plots [positive **(A)** and negative **(B)** ion mode] displaying grouped discrimination among the three groups, based on the first two principal components. **(B)** Loading plots of PLS-DA model [positive **(A)** and negative **(B)** ion mode] derived from the potential biomarker metabolites in the serum samples of normal, model, and ZWT groups. **(C)** Top 10 important discriminatory metabolites upon ZWT treatment, as identified using KEGG pathway enrichment analysis. **(D)** Schematic overview of bile secretion metabolic pathway changes in IgAN rats upon ZWT treatment. The metabolites are shown in color: red color represents an increase in the levels of metabolite, while green color represents a decrease.

The metabolomic profiles of serum samples in each group displayed satisfactory separation based on the PCA model [positive (a) and negative (b) ion mode] (Figure 6A). A PLS-DA model [positive (a) and negative (b) ion mode] was established to illustrate the metabolic distinction between the three groups, which suggested that upon treatment with ZWT, the IgAN group tended to approach the status of the normal group (Figure 6B). 784 (positive ion mode) and 647 (negative ion mode) markedly altered metabolic species were selected (VIP > 1, *p* < 0.05) using multivariate statistical analysis of serum samples from ZWT-treated rats (Supplementary Table S7). These biomarkers of serum metabolites regulated by ZWT were mainly involved in carbohydrate metabolism, amino acid metabolism, metabolism of other amino acids, biosynthesis of other secondary metabolites, lipid metabolism, and metabolism of cofactors and vitamins. Metabolic pathway analysis was performed to obtain an overall view of the above metabolic biomarkers and their potentially functional effects (Figure 6C). The top ten metabolic pathways including HIF-1 signaling pathway, bile secretion, pentose phosphate pathway, and aldosterone synthesis/secretion were then finalized based on *p* values. The exhaustive KEGG map of bile secretion was taken as a representative example (Figure 6D).

#### Histopathological and Biochemical Analysis in Immunoglobulin A Nephropathy Rats upon Zhen Wu Tang Treatment

**FIGURE 7:**
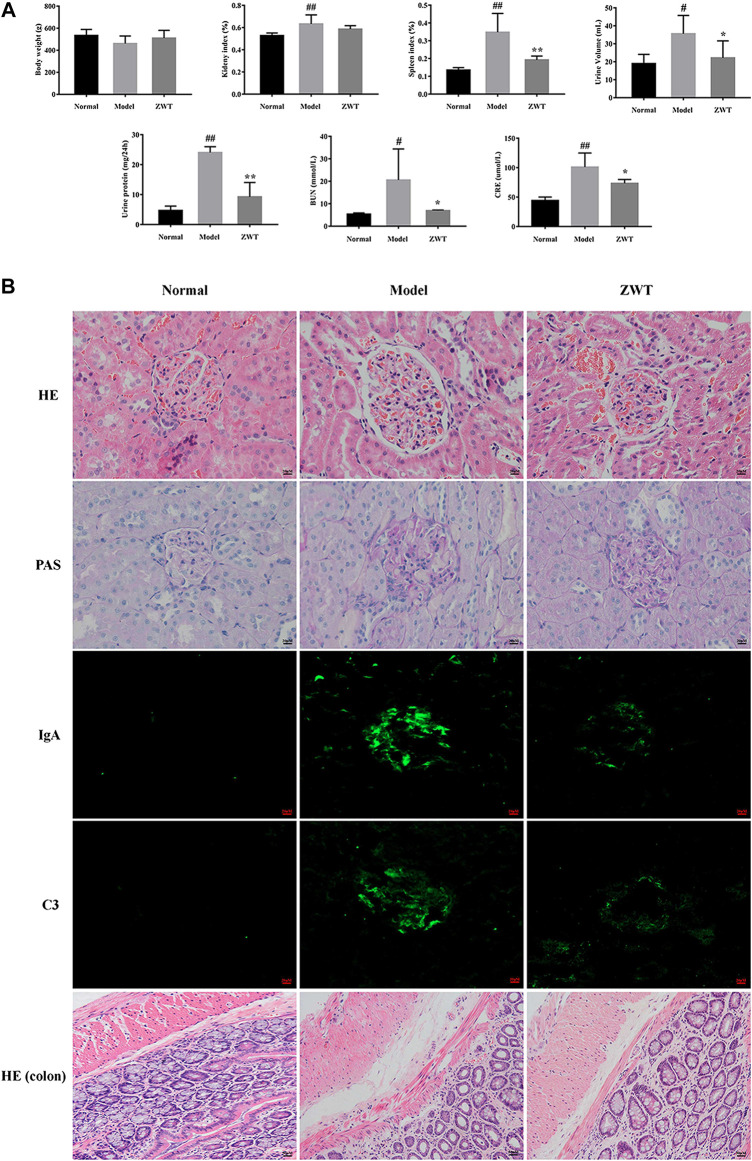
Effect of ZWT on kidney function and colonic injury. **(A)** Effect of ZWT on body weight, kidney index, spleen index, and biochemical parameters including urine volume, 24-h urine protein, BUN, and CRE. **(B)** Photomicrographs of HE staining (400×), PAS staining (400×), and immunofluorescence staining (200×) of IgA and C3 immune complexes in glomeruli of renal tissues and HE staining (200×) of colon tissues in IgAN rats. Values represent mean ± SD, n = 5. Vertical bars represent standard deviation of the mean. ^#^ *p* < 0.05, ^##^ *p* < 0.01 vs. normal; *p < 0.05, **p < 0.01 vs. model.

As shown in Figure 7A, the IgAN model rats exhibited decreased body weight, increased BUN, CRE, urine volume, and 24-h urine protein, as compared to rats in the normal group. Furthermore, compared to the normal group, renal pathological investigation in IgAN rats exhibited evident glomerulus swelling, proliferation of mesangial cells, and extension of mesangial matrix; interestingly, treatment with ZWT remarkably reversed these morphological changes. PAS staining of renal tissues of IgAN rats revealed obvious deposition of immune complexes in the mesangial areas, an effect that was significantly inhibited upon ZWT treatment. Similarly, the markedly increased deposition of IgA and C3 immune complexes observed in the kidney tissue of the model group was attenuated in the ZWT-administered group. In addition, compared to the normal group, histology of the intestinal mucosa of the model group displayed abnormal morphology, structural disorder, and inflammatory infiltration. The pathological change in the model group was significantly alleviated upon ZWT therapy (Figure 7B). Taken together, the above results demonstrated that treatment with ZWT could mitigate renal pathological damage and attenuate kidney function injury and its consequences in IgAN rats.

## Discussion

Gut microbiota is a complex microbial ecosystem of the human body. Differences in the composition and structure of the gut microbiota are associated with the onset and development of multiple diseases (Qin et al., 2012; Bajaj, 2019; Singh et al., 2020). Accumulating studies have emphasized the essential roles of the gut–kidney axis and the bidirectional relationship between the host and the gut flora in humans and animals (Zhang et al., 2018). Additionally, recent evidence suggests that an abnormal profile of gut microbiota and changes in the metabolic phenotype are closely linked to both patients and animals with CKD (Nicholson et al., 2012; Wang et al., 2020). In the present study, 16S rDNA gene sequencing analysis of fecal or serum samples, in combination with the untargeted metabolomics approach, provided comprehensive insights into the association between IgAN and intestinal microbiota and related metabolite alterations. In addition, we further demonstrated that the effect of ZWT on ameliorating kidney injury might be mediated through adjustments in the intestinal flora and restoration of metabolism homeostasis.

According to a previous study, there is an adverse change in the gut microbiota in IgAN patients, as compared to healthy individuals. In the present study, fecal samples of IgAN model rats showed abnormal changes in the gut microbial community, characterized by altered diversity and differences in bacterial population structure and composition, as compared to the normal rats. Post establishment of the IgAN model, significant alterations in the richness of 51 bacterial species including *Lactobacillus*_*reuteri*, Rikenellaceae_RC9_gut_group, Ruminiclostridium_9, Actinobacteria, and Bifidobacterium were observed in the IgAN rats, highlighting the profound influence of IgAN on disturbance of intestinal flora. Result of gut microbiota analysis showed that, compared to the normal group, the IgAN model group presented distinct degrees of disorder (from the phylum to species levels). Treatment with ZWT ameliorated this microbial dysbiosis, indicating that ZWT plays a role in regulating gut microbiota. We found Firmicutes and Bacteroidetes to be the most dominant bacterial phylum in the fecal samples, consistent with other studies. We also found enrichment of Ruminococcaceae_NK4A214_group, Ruminococcaceae_UCG-014, and depletion of Enterorhabdus and Coriobacteriaceae_UCG-002 in the normal rats at the genus level. In addition, at the bacterial family level, increasing abundance of Lachnospiraceae and Burkholderiaceae were observed in the IgAN rats, with a decrease in Muribaculaceae and Christensenellaceae. In light of the above comprehensive results, we confirmed the concurrence of microbial dysbiosis in IgAN rats. Difference in gut microbiota has the potential to serve as a biomarker target for guiding noninvasive diagnosis. Promising treatments can target amelioration of IgAN-induced microbial dysbiosis. Given that the diagnosis of gut microbiota is influenced by all kinds of factors, the biomarker is not a single bacterium but a set of bacteria. The reliability of these bacteria as biomarkers needs to be explored in future studies. Hence, large-scale prospective studies with extensive number of samples from IgAN patients and mice need to be conducted to identify and develop more reliable microbiome biomarkers.

The pathogenesis of IgAN is multifactorial and involves the interaction of host genetics, immune disorder, and environmental elements to bring about an abnormal immune response and chronic inflammation. Although studies on the complex association between kidney tissue damage and gut have been carried out over time, the underlying mechanism still remains unknown. Based on the theory of the kidney–gut axis, many studies have confirmed that kidney disease is related to intestinal disorders *via* alterations in the composition of the intestinal flora and the generation of uremic toxins (Chen et al., 2019). Although the primary cause for IgAN is chronic inflammation and immune disorders, growing evidence links the pathogenesis of IgAN to the dysregulation of gut microbiota (Hu et al., 2020). Normal intestinal flora plays a decisive role in the development of diseases as it mediates environmental changes in the body’s immune system and modulates the chronic inflammatory state. Previous studies have also shown that CD4^+^ T cells can engage in crosstalk with the gut flora to participate in immune diseases during inflammation (Brown et al., 2019). Particularly, with a key role in immune response, CD4^+^ T cells can affect IgAN evolution and progression (Bai et al., 2019a). Initial observations in IgAN rats have shown decreased proportion of Anaerovorax and increased proportion of Enterobateriales and *Escherichia shigella*, as compared to normal rats. Proliferation of gram-negative bacteria (with a typical genus of *Escherichia shigella*) and leakage of LPS may trigger a severe inflammatory state in the IgAN group. In a previous study, we discovered that injury of mesangium cells is induced by a stimulus (LPS), which could increase the release of inflammatory cytokines and arouse an inflammatory milieu (Bai et al., 2019b). Accordingly, IgAN-associated systemic inflammation may be associated with the intestinal flora by mechanisms involving bacterially derived LPS. In addition, decrease of Anaerovorax, the SCFA-generating bacteria, is related to increased renal inflammation and injury of the intestinal mucosal barrier. SCFAs including butyric acid, propionic acid, and acetic acid exert crucial roles in peripheral T cell differentiation, thereby modulating inflammation (Huang et al., 2017). Consequently, gut flora may act as a new biomarker for the treatment of chronic diseases, including inflammation and dysimmunity.

IgAN is characterized by a state of chronic, low-grade, systemic inflammation due to immune disorders. A large body of proof has indicated that inflammation exerts an indispensable effect on renal damage and progression of IgAN. In this study, HE staining revealed massive accumulation of inflammatory cells in the renal and colonic tissues of IgAN rats. As direct indicators of inflammatory response, upregulation of IL-17, IL-4, and IL-1β in the renal tissues of IgAN rats has been reported in our previous study; however, the proposed mechanism did not take gut microbiota into consideration (Bai et al., 2019a). Since microbes are reported to be related to inflammatory cytokines, changes in microbes may play a pivotal part in the inflammatory status of renal damage, and the same must be further investigated. Moreover, the anti-inflammatory role of ZWT has been reported in a previous study. Liu et al. (2019) found that ZWT significantly suppressed the activation of the NLRP3 inflammasome, reduced the protein expression of IL-1β as well as the release of IL-18. ZWT significantly decreased the renal phosphorylation of NF-κB but increased the expression of IκB in IgAN rats (Liu et al., 2018). These studies link the anti-inflammatory mechanism of ZWT with gut microbes under the condition of IgAN. Our results revealed the potential anti-inflammatory mechanism of the intestinal microbiome in IgAN upon ZWT treatment, which deserves detailed experimental and clinical investigation in the future.

In addition to the gut flora in the feces, serum metabolites may also reflect the physiological status of an individual. Data in the present study showed that there was an obvious increase in the renal function of IgAN rats upon ZWT treatment, which might be due to its anti-inflammatory effect; however, the exact biochemical mechanism responsible for this effect needs further study. LC-MS-based metabolomics analyses are being increasingly performed to explore the abnormality of serum metabolites in multiple diseases. The present study was designed to obtain comprehensive understanding of the changes in the serum metabolome and to explore latent targets in IgAN rats and their response to treatment with ZWT. In the present study, the untargeted metabolomics approach was applied to identify serum metabolic profiles related to IgAN, followed by evaluation of the effect of ZWT on IgAN. Identified serum metabolites were relevant to the dysbiosis of metabolism, chiefly involving processes such as lipid metabolism, signal transduction, carbohydrate metabolism, amino acid metabolism, and biosynthesis of other secondary metabolites, consistent with the potential function of gut flora. As shown in Figure 5C, treatment with ZWT could regulate various metabolic pathways in IgAN rats, including HIF-1 signaling pathway, bile secretion, pentose phosphate pathway, and aldosterone synthesis/secretion. Our study further demonstrated that treatment of IgAN rats with ZWT resulted in intervention in some metabolic pathways, possibly achieved by restoring the changes in some targets discovered in this investigation, to prevent the disruption of the intestinal epithelial barrier. Breakdown of the gut epithelial barrier triggers local and systemic inflammation as well as the influx of leukocytes, further accelerating the translocation of uremic toxins into the systemic circulation in CKD patients and animals. In addition, it has been reported that altered gut microbiota is the reason for excessive generation of uremic toxins (e.g., indoxyl sulfate, *p*-cresyl sulfate, and trimethylamine-N-oxide) and a decrease in renoprotective metabolites (Tang et al., 2015). The microbial balance in the gut is perturbed following IgAN exposure, which can lead to over accumulation of uremic toxins, further promoting the release of pro-inflammatory factors. As a consequence of long-term stimulation of inflammation, there is a deterioration of kidney function and further aggravation of tissue damage.

Renal function is usually reflected in the levels of CRE, BUN, and 24-h protein urine. Increased deposition of IgA complexes in the kidney tissue is a strong sign of IgAN. Results of the histopathological examination conducted in this study were consistent with those obtained in earlier studies, suggesting that the IgAN model rat was successfully established. We believe that ZWT ameliorated impaired kidney function and decreased the deposition of IgA complexes by partially reversing the metabolic aberrance in the serum and recovering the dysregulation of gut microbiota. As shown in Figure 7B, pathological changes, including crypt damage accompanied by edema and irregularly arranged goblet cells, were observed in the gut of IgAN rats. It has been reported that disruption of the intestinal epithelial barrier contributes to increased serum cytokines levels and renal impairment in IgAN. In the present study, increasing abundance of Collinsella was observed in the IgAN rats. A previous study has demonstrated that Collinsella can enhance gut permeability by lowering expression of tight junction proteins, resulting in an altered immune status and local inflammation (Chen et al., 2016). Consequently, it is reasonable to speculate that higher richness of Collinsella may control the intestinal mucosal barrier function through the above mentioned mechanisms.

In conclusion, in the present study, we performed 16S rDNA sequencing and LC/MS detection to characterize the structure of the gut flora in fecal samples, analyze its abundance, and evaluate the relation between IgAN, gut microecology, and related serum metabolites. The data obtained in the research might provide a biological basis for comprehension of the mechanism underlying the relationship between gut flora, serum metabolites, and IgAN, in addition to providing new insights into the therapeutic mechanism of ZWT. Moreover, intestinal flora and related metabolites serve as biomarkers in kidney disease, changes in which can reflect the stress response to IgAN; thus, such biomarkers display the potential to guide clinical diagnosis and treatment.

## Data Availability Statement

The datasets presented in this study can be found in online repositories. The names of the repository/repositories and accession number(s) can be found in the article/Supplementary Material.

## Ethics Statement

The animal study was reviewed and approved by Animal Ethics Committee of Guangzhou University of Chinese Medicine.

## Author Contributions

JL and HL wrote the original manuscript; JL, HL, YC, and RL designed and performed the study; HF, GF, and QC performed analysis and interpreted the data; YC and QC visualization; BL and JW revised the manuscript; YZ and JZ supervised study and funding acquisition.

## Funding

This research was supported by the National Natural Science Foundation of China (Grant No. 81673874, 81803824, and 81603371) and the Natural Science Foundation of Guangdong Province China (Grant No. 2018A030313328 and 2018B0303110004).

## Conflict of Interest

The authors declare that the research was conducted in the absence of any commercial or financial relationships that could be construed as a potential conflict of interest.
